# Making the Right Diagnosis: Slowly but Surely

**DOI:** 10.1161/CIRCULATIONAHA.123.067074

**Published:** 2023-11-27

**Authors:** Parag R. Gajendragadkar, Claire A. Martin, Rohan S. Wijesurendra

**Affiliations:** Department of Cardiology, Royal Papworth Hospital, Cambridge, United Kingdom (P.R.G., C.A.M.).; Nuffield Department of Population Health, University of Oxford, United Kingdom (P.R.G., R.S.W.).; Division of Medicine, University of Cambridge, United Kingdom (C.A.M.).; Department of Cardiology, John Radcliffe Hospital, Oxford, United Kingdom (R.S.W.).

## ECG CHALLENGE

**Figure 1.**
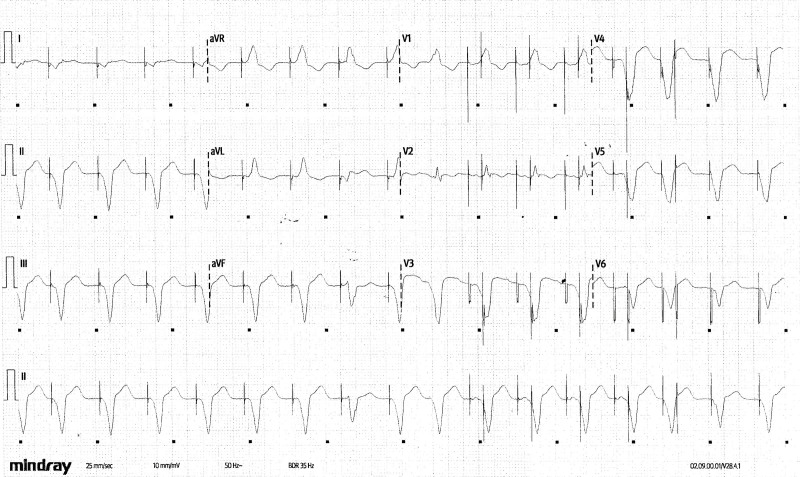
ECG on admission.

An 84-year-old man with severe ischemic cardiomyopathy and a dual chamber implantable cardioverter defibrillator with previous treatment for sustained ventricular arrhythmias presented after feeling generally unwell for 4 days. There was no history of chest pain, dyspnea, palpitation, or syncope. On examination, he was alert and oriented, afebrile, and with a blood pressure 110/70 mm Hg. The remainder of the clinical examination was normal with no evidence of fluid overload. The ECG shown in Figure 1 was recorded, and he was admitted to the ward for further investigation and treatment.

What is the diagnosis?

Please turn the page to read the diagnosis.

## RESPONSE TO ECG CHALLENGE

**Figure 2.**
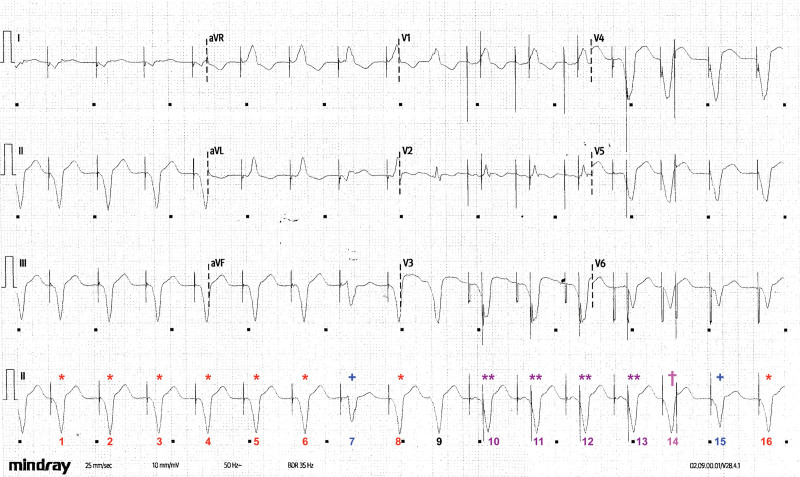
**Annotated ECG of presenting rhythm.** Corresponding to Figure 1. Complexes 1 to 6 and 16 (*) correspond to *atrial* pacing spikes followed by a sensed intrinsic ventricular rhythm or “slow VT” (which appropriately inhibits ventricular pacing). Complexes 7 and 15 (+) correspond to *atrial* pacing spikes followed by slightly different ventricular complexes, indicating an alternative exit site from the “slow VT” circuit. Complex 9 shows no pacing but unabated intrinsic ventricular rhythm; this confirms the diagnosis of “slow VT.” Complexes 10 to 13 (**) show atrial pacing with P waves followed by ventricular pacing spikes, but there is no change in the morphology of the ventricular complexes, confirming that this is still “slow VT” with no pacing capture (pseudofusion). Complex 14 (†) shows an *atrial* pacing spike (with no P wave), but in this case, the intrinsic ventricular rhythm is not sensed, because it falls straight after the atrial pacing spike, so a redundant ventricular pacing spike is delivered early within the T wave. VT indicates ventricular tachycardia.

Figure 1 (annotated and reproduced in Figure 2) shows a regular broad complex rhythm at 96 bpm. QRS complexes 7 and 15 have a different morphology from the others. At first glance, pacing spikes seem to precede the QRS complexes. However, complex 9 shows that the rhythm is independent of the presence of pacing. P waves are not seen consistently, although atrial pacing spikes and P waves are seen before complexes 10 to 13.

**Figure 3.**
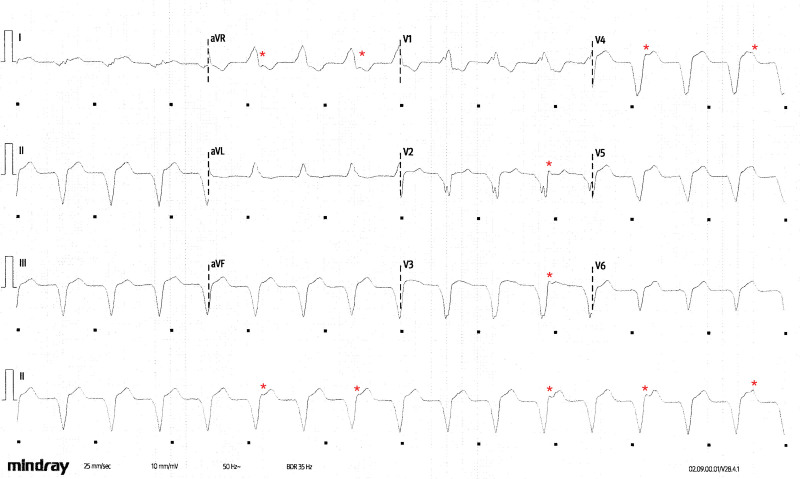
**ECG with pacing disabled.** Annotated ECG recorded with pacing temporarily disabled, demonstrating an ongoing ventricular rhythm with independent atrial activity (*) and confirming the diagnosis of “slow ventricular tachycardia.”

This broad, complex rhythm, which is independent of pacing and with atrioventricular dissociation, is a ventricular rhythm—essentially ventricular tachycardia (VT), but at less <100 bpm (sometimes referred to as “slow VT”). Inhibition of pacing confirmed the diagnosis, with evidence of atrioventricular dissociation and independent regular atrial activity (marked with an asterisk; Figure 3).

**Figure 4.**
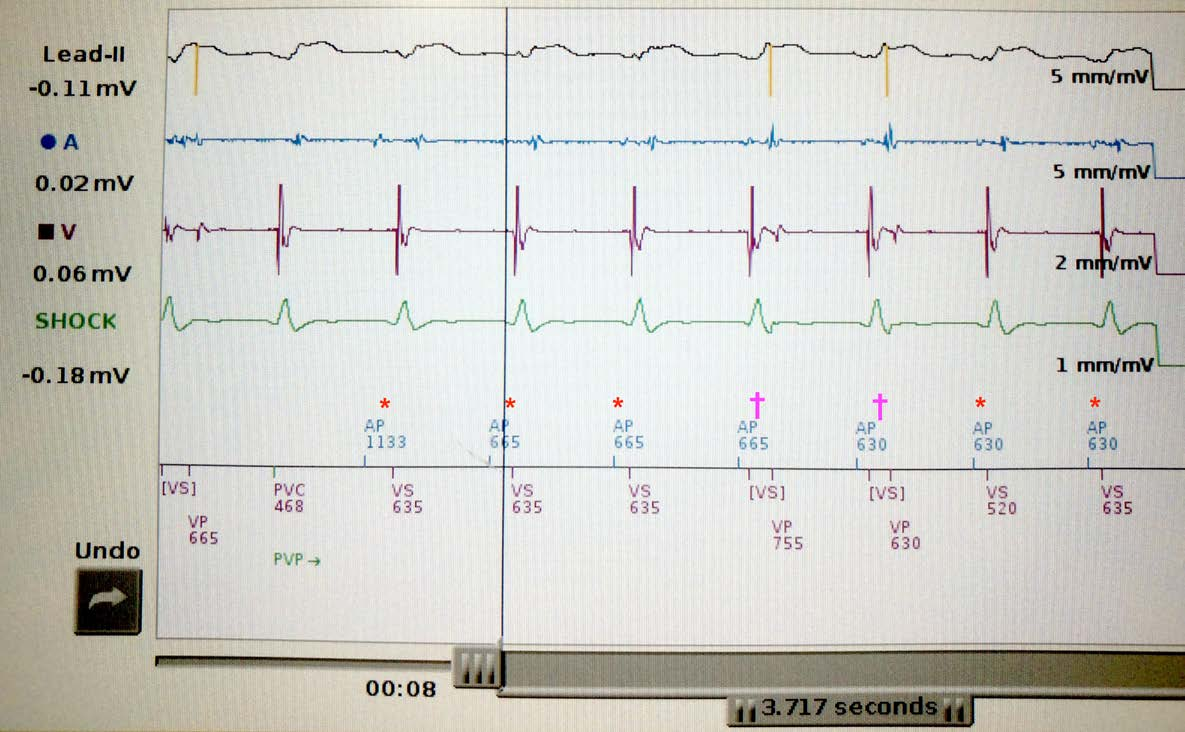
**Device interrogation.** Screenshot of device interrogation during “slow VT.” From top to bottom: ECG lead II showing broad complex tachycardia; “A” (atrial) electrogram channel; “V” (ventricular) electrogram channel; “Shock” as coil to can pseudo-ECG; device markers. The asterisk (*) shows atrial pacing (AP) with subsequent ventricular sensing (VS) corresponding to the 12-lead ECG appearance of an AP spike followed by an intrinsic QRS. The dagger (†) shows AP with VS. The VS is ignored by the device because of its occurrence straight after the AP spike (in a “blanking period”), and hence attempted ventricular pacing (VP) occurs; this can be seen on the surface ECG to fall within the QRS. The label PVC refers to a “premature ventricular complex”—ie, the device interprets this intrinsic ventricular electrogram occurring “early” after a VP event as a PVC. In fact, the rhythm throughout this screenshot is “slow VT.” VT indicates ventricular tachycardia.

During the original ECG recording, the VT rate was just above the programmed device base rate. Device interrogation showed that all parameters were within normal limits and confirmed *atrial* pacing spikes coinciding with the intrinsic ventricular rhythm (Figure 4). This is therefore pseudo-pseudofusion,^1^ where an intrinsic QRS is overlapped with an *atrial* rather than a ventricular pacing output, giving the appearance of pseudofusion (where a ventricular pacing spike is followed by a noncaptured intrinsic QRS). The patient had recently previously presented in a similar rhythm of “slow VT” at 90 bpm, and the device base rate had been increased to 95 bpm in an attempt to overdrive suppress the arrhythmia while awaiting a planned ablation procedure.

### Clinical Progress

**Figure 5.**
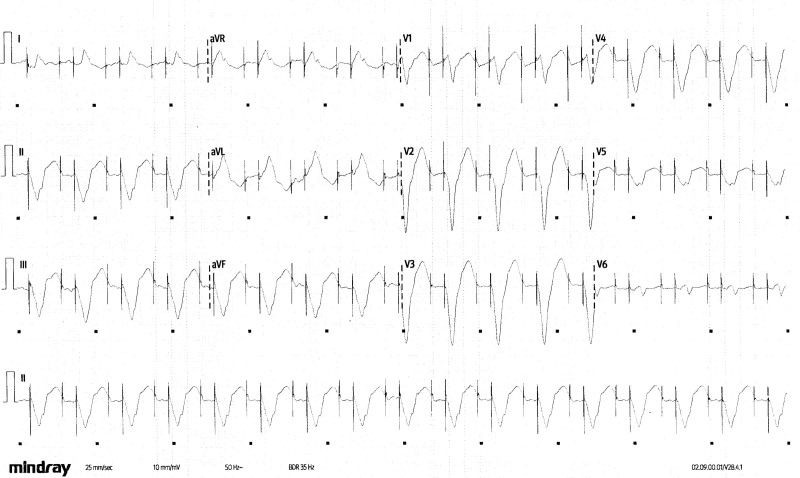
**ECG after reprogramming for pacing.** ECG after reprogramming the device to a base rate of 100 bpm. This shows dual chamber atrioventricular pacing. The morphology of the ventricular paced beats is different from the ventricular beats on previous ECGs, again confirming that the broad ventricular complexes seen on the previous ECGs were caused by an intrinsic ventricular rhythm (“slow ventricular tachycardia”) rather than ventricular pacing.

**Figure 6.**
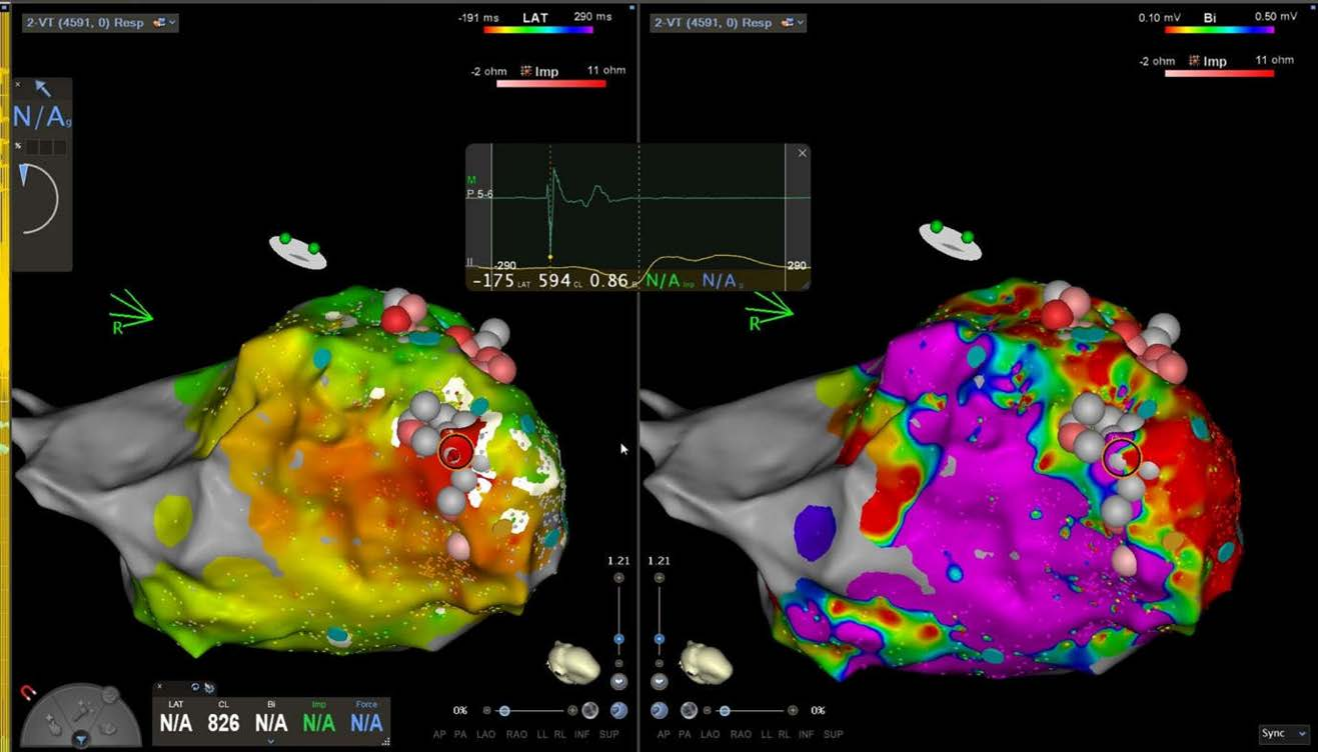
**Endocardial 3-dimensional map of the left ventricle during ablation.** Activation timing is shown on the **left**, and voltage is shown on the **right**; spheres represent ablation lesions. The highlighted dark red circle shows the point of slow conduction in a scar border zone where ablation terminated the arrhythmia, confirming the re-entrant mechanism. Maps created using the CARTO 3D mapping system.

A further increase in the pacing base rate to 100 bpm restored conventional dual chamber sequential pacing and restored atrioventricular synchrony (Figure 5). An urgent inpatient VT ablation was organized. Invasive electrophysiological study confirmed the presence of a large anterior left ventricular scar. The “slow VT” was induced and mapped endocardially; middiastolic potentials were identified at an inferoapical point in the scar border zone, and ablation here terminated the arrhythmia (Figure 6). This confirmed a re-entrant mechanism consistent with ventricular tachycardia (versus an automatic idioventricular rhythm).

## ARTICLE INFORMATION

### Sources of Funding

None.

### Disclosures

None.

## References

[1] Levine PA Seltzer JP. Fusion, pseudofusion, pseudo-pseudofusion and confusion: normal rhythms associated with atrioventricular sequential “DVI” pacing. Clin Prog Pacing Electrophysiol. 1983;1:70–80. doi: 10.1111/j.1540-8167.1983.tb01599.x 10.1111/j.1540-8159.1983.tb05303.x 6191300

